# Evaluating the advantages of passive exoskeletons and recommendations for design improvements

**DOI:** 10.1177/20556683241239875

**Published:** 2024-03-21

**Authors:** Sajid Rafique, Shaikh Masud Rana, Niclas Bjorsell, Magnus Isaksson

**Affiliations:** 1 Department of Electrical Engineering, Mathematics, and Science, 101068 University of Gävle, Gävle, Sweden; 2 Faculty of Health and Occupational Studies, 560571 University of Gävle, Gävle, Sweden

**Keywords:** Evaluation of exoskeletons, musculoskeletal disorders, EMG sensor, muscle activity, drilling, assistance for workers

## Abstract

Construction and manufacturing workers undertake physically laborious activities which put them at risk of developing serious musculoskeletal disorders (MSDs). In the EU, millions of workers are being affected by workplace-related MSDs, inflicting huge financial implications on the European economy. Besides that, increased health problems and financial losses, severe shortages of skilled labor also emerge. The work aims to create awareness and accelerate the adoption of exoskeletons among SMEs and construction workers to reduce MSDs. Large-scale manufacturers and automobile assemblers are more open to adopt exoskeletons, however, the use of exoskeletons in small and medium enterprises (SMEs) is still not recognized. This paper presents an experimental study demonstrating the advantages of different exoskeletons while performing workers’ tasks. The study illustrates how the use of certain upper and lower body exoskeletons can reduce muscle effort. The muscle activity of the participants was measured using EMG sensors and was compared while performing designated tasks. It was found that up to 60% reduction in human effort can be achieved while performing the same tasks using exoskeletons. This can also help ill workers in rehabilitation and putting them back to work. The study concludes with pragmatic recommendations for future exoskeletons.

## Introduction

Majority of workers experience pain from MSDs due to strained or aggravated muscles, joints, nerves, discs, ligaments and tendons. MSDs are commonly caused by repetitive arduous motions over a longer period of time. Therefore,consistent MSDs can impact the health of a person on long term basis. In the US, according to the Bureau of Labor Statistics (BLS), majority of the work-related injuries are occurring due to MSDs, as almost 30% of all workers’ compensations were reported due to MSDs.^1^ Therefore, MSDs inflicting US companies with exuberating economic costs and according to the United States department of labor, US companies spent almost 300 billion dollars annually on direct and indirect costs owing to the sicknesses related to MSDs.^1,2^ Similarly, EU economies have also been suffering with heavy losses owing to workplace-related MSDs, which is inflicting more than €240 billion annually to the European economy.^3–5^

To alleviate the detrimental impacts of work-related MSDs in physically strenuous jobs, research is now focusing on developing new assistive techniques, such as the use of robotic exoskeletons. An exoskeleton is a wearable device that increases and supports human movement with lesser energy, thereby reducing the wearer’s physical effort, which in turn could minimize the risk of developing work-related MSDs.^6^ Typically, exoskeletons can be classified as either active or passive.^7^ Active systems consist of one or more powered actuators to increase the strength of the human being,^8–10^ whereas passive systems use material compliance and spring/elastic members to store and release energy during activities to help employees make physical movements.^11,12^ The term ‘wearable robotics’ came into existence in 1960s when research efforts started to focus on developing load augmentation systems for military and rehabilitation.^13^ Subsequently, interest continues to grow with new innovations reported regularly. For specific military uses, a limited number of active exoskeletons have been developed such as BLEEX and Sarcos XOS2 to improve soldier’s physical strength or carrying capacity.^14,15^ Active exoskeleton systems are more complex and provide higher support, whereas, the passive exoskeleton systems are much simpler but provide lower assistance. The passive exoskeletons are much cheaper and can be used without any supervision and are quite useful for jobs involving frequently handling of moderate loads. Unlike SMEs, some of the leading automobile companies such as BMW, Ford, Honda, Nissan, Toyota, and Volkswagen have adopted the use of passive exoskeletons and there are about 585 exoskeleton devices in use which are helping to reduce MSD related injuries of their workforce.^16^ It is demonstrated that a quality improvement of 86% was observed in a high quality welding while using a shoulder support passive exoskeleton Levitate Airframe.^16^ This comparison was made while performing the same welding task with and without wearing the exoskeleton.

However, with respect to other industrial sectors such as small and medium enterprises (SMEs) and construction sector, the idea of exoskeleton is relatively new and as such is still finding difficulties in adoption among SMEs.^4–6^ Therefore, the broader use of such exoskeletons among SMEs, logistics and construction sector is still unknown due to lack of acquaintance with assistive technology.^10^ Also, smaller companies lack the financial means or awareness to invest in such advancements. Additionally, large corporations often have a higher risk tolerance and can absorb potential setbacks more easily, fostering a culture of innovation.^10^ The cost-to-benefit assessments of adopting passive exoskeletons in relation to sickness compensation can vary. While initial costs for implementing exoskeletons may be high, the potential benefits include reduced worker fatigue, lower injury rates, and increased productivity. Over time, these advantages could lead to cost savings through fewer sick days and workers' compensation claims.^10^ It’s essential for companies to assess the long-term impact on employee health and overall operational efficiency when considering such investments.

Owing to this reason, this paper explores the advantages of using exoskeletons while performing different tasks of workers of SMEs, construction and logistic sector to illustrate directly the advantages of exoskeletons. The work presented in this paper was conducted under North Sea Region’s EXSKALLERATE project at the University of Gavle, Sweden. The main objective of EXSKALLERATE was to accelerate the adoption of exoskeletons into construction and industrial manufacturing SMEs, where heavy physical work leads to severe health issues, and thereby, strengthen SME competitiveness in the North Sea Region.^17^ The project aimed to provide the workers of SMEs a real-time experience of using exoskeletons at their workplaces by setting up end-user pilots sites. This experimental study is part of EXSKALLERATE and tackles these issues and explains a viable solution which can reduce economical and health damages caused by MSDs. It is pertinent to mention that in EXSKALLERATE project, a few insurance companies dealing with the work-related injuries in the EU also showed their strong interests in adoption of exoskeletons and envisaged that certain exoskeletons will be part of the essential gear of workers in future. As part of EXSKALLERATE project, a work^17^ recently presented the experimental assessments of using exoskeletons for workers. The work reported a reduction in muscle activity of participants while using an upper limb exoskeleton and performing overhead assembly, bricklaying, and box moving tasks. However, the work^17^ did not record and present the muscle activity data of workers while performing their tasks. This study presents the muscle activity of participants measured using EMG sensors.

This work presents the functioning of upper and lower body exoskeletons and illustrates the importance of choosing the correct exoskeleton to perform a task. It is important to note that the body posture to perform a worker’s specific task shall synchronize with the designed range of motions (ROMs) of an exoskeleton to garner maximum support. Therefore, accurate selection of the exoskeletons for a specific type of work, can reduce human efforts significantly which can alleviate the stresses on the muscles and joints of the worker. On the other hand, if the exoskeleton and the human body posture are not synchronized to perform a certain task, it will increase the stresses on muscles and joints, and can have detrimental effects.

### A brief Survey of passive exoskeletons for workers

There are a number of upper and lower body passive exoskeletons available in the market, costing around $5000 to $8000 with an average working life of 3 to 5 years as shown in Table 1. Every exoskeleton is particularly designed to perform specific ROMs and, hence, provide optimum support at particular body position.^12–19^ For example, upper body passive exoskeleton, EksoVest, developed by Eksobionics is suitable to perform overhead and shoulder level repetitive tasks such as in the assembly line of automobiles.^18^ Therefore, EksoVest is not suitable to perform tasks which involve frequent bending and lifting of things from the ground. Similarly, for virtual chair position and deep squatting positions, another passive exoskeleton, such as LegX, developed by SuitX was found to be more appropriate as it specifically targets to provide assistance at these positions.^19^ Owing to these facts, it is important to analyze the ROMs, body positions and loading requirements prior to selecting any exoskeleton for a particular task. Table 1 below shows a brief list of available exoskeletons along with a description of their suitability of performing different tasks.

**Table 1.** Survey of some existing upper and lower body commercial exoskeletons for workers.

	Name of Exo	Suitable tasks	Manufacturer	References
Upper Body Passive Exoskeletons for workers	EksoVest Or EVO	Overhead & shoulder level tasks	Ekso Bionics	^18^
	Levitate Airframe	Upper body tasks	Levitate technologies	^20^
	V3 ShoulderX	Overhead & shoulder level tasks	SUIT-X	^21^
	FLX, and the V22	Bending and lifting	StrongArm technologies	^22^
	Laevo	Forward bending	Laevo	^23^
	ATOUN	Bending and lifting	ATOUN Inc	^24^
	Muscle Suit	Bending and lifting	Innophys	^25^
	HA EXO-O1	Overhead & shoulder level tasks	HILTI	^26^
	Paexo shoulder	Overhead & shoulder level tasks	Ottobock Bionic	^27^
	MATE-XT	Upper body exoskeleton	MATE	^28^
	VEX	Upper body exoskeleton	Hyundai	^29^
	*Skelex*	Upper body exoskeleton	Skelex	^30^
	*HAL* *Lumber Support*	Upper body exoskeleton	HAL	^31^
Lower body passive exoskeletons for workers	LegX	For virtual chair and kneeling position	SUIT-X	^19^
	Chairless chair	Virtual chair position	Noonee	^32^
	Archelis chair	Virtual chair position	Archelis	^33^
	ExoChair	Virtual chair position	TADVISER	^34^
	CEX	Lower body tasks	Hyundai	^35^

### Other areas of applications of passive exoskeletons

The main objective of EXSKALLERATE was to accelerate the adoption of exoskeletons into construction and industrial manufacturing SMEs, where heavy physical work leads to severe health issues, and thereby, strengthen SME competitiveness in the North Sea Region. Apart from having advantages for the workers, passive exoskeletons have shown their potential in different areas aiming to enhance human performance, reducing the risk of injuries, and improving the quality of life for users. For example, in elderly assistance, the global population of persons aged over 65 years was 727 million in 2020 which, over the next three decades, is projected to more than double, reaching over 1.5 billion in 2050.^36^ This will exert huge pressure on the health care sector and, hence, exploring the applications of passive exoskeletons to support the elderly might have tremendous potential for growth. There exist few passive exoskeletons which were tested for elderly assistance such as EksoGT, The Chairless chair and Soft Exossuit.^37^

The application of passive exoskeletons is also being explored for rehabilitation and physical therapy.^38^ Passive exoskeletons are used in rehabilitation centers and clinics to aid individuals recovering from injuries or surgeries. These exoskeletons provide support to specific body parts, such as the lower limbs or the upper extremities, helping patients regain mobility and strength during the rehabilitation process.^38^ However, the focus of research in this area has been more on the development of active exoskeletons due to the need for a higher level of assistance. Passive exoskeletons are also being explored in sports and athletics to enhance performance and prevent injuries. Athletes may use exoskeletons during training to support specific muscle groups or joints, improving biomechanics and reducing the risk of overuse injuries.^39^ Some of the examples of exoskeletons being used in sports are Againer, Elevate, RoboGolfPro, Ski-Mojo, and Xnowers.^39^

Another study^40^ showed that passive exoskeletons have potential applications in space exploration, where astronauts may benefit from assistance with mobility and strength in the microgravity environment of space. These exoskeletons could aid astronauts during extravehicular activities (EVA) or when working in confined spaces within spacecraft or space stations. In a study, A wearable passive exoskeleton is proposed for reduced gravity astronaut training.^40^ The main component unit of the proposed robotic exoskeleton is the spring-based parallelogram mechanism which can passively balance any proportion of the gravity load acting on it by designing an appropriate stiffness of the spring or adjusting the install position of the spring.^40^

### Structure of the paper

The paper is organized as follows: Section 2 describes the experimental setup containing a description of the hardware/software, the two exoskeletons used, tasks performed, participants selection, ethical approval, data collection and analysis. Results are presented in Section 3 and Section 4 states the design recommendations for future industrial exoskeletons. Finally, Section 5 concludes the paper by summarizing the conclusion of the study.

## Experimental setup

The study aims to measure and compare muscle activity of the participants, with and without wearing exoskeletons, while performing different tasks of workers. These tasks mainly involved overhead, shoulder level, kneeling and virtual chair position postures. In this study, the EMG sensors were attached around the shoulder and leg at biceps/triceps brachialis and biceps femoris, respectively, to observe the muscle activation while performing different tasks of workers at different positions such as Roof drilling, Wall drilling, lifting/shifting of loads, virtual chair position and knee position. The surface electrodes of EMG Sensors measured the EMG signal associated with muscle contractions, muscle response, and activation level.

### Exoskeletons used in this study

**Figure 1.**
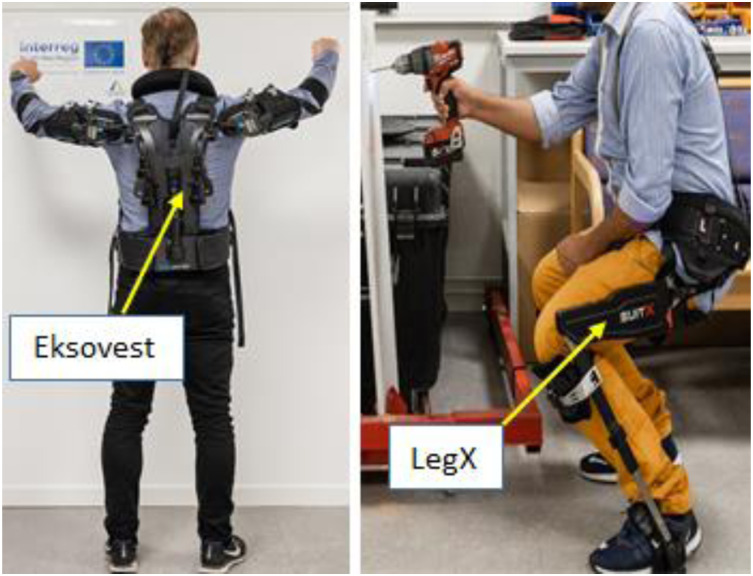
Eksovest and LegX.

The aim of the study was to demonstrate the performance of exoskeletons involving upper and lower body. Therefore, EksoVest manufactured by Eksobionics was used to perform tasks involving the upper body, whereas, LegX developed by SuitX was used to perform tasks involving virtual chair position and deep squatting positions as shown in Figure 1, respectively. These exoskeletons were chosen for this study as Eksobionics and SuitX are the leading passive exoskeleton companies which are offering technologies that enhance worker’s capabilities globally. Moreover, both exoskeletons offer the required ROMs needed to perform the selected tasks of this study. The results compared human efforts and muscle activities while undertaking different tasks with and without wearing these exoskeletons. The salient features of Eksovest and LegX exoskeletons are summarized below,

#### Eksovest

Eksovest is suitable for overhead and shoulder-level tasks. The weight of the Eksovest is 4.3 kg. There are four spring support levels in Eksovest to adjust four different payloads. The support levels are L1 = 2.2 - 3.1 Kg, L2 = 3.1 - 4.0 Kg, L3 = 4.0 - 5.4 Kg, L4 = 5.4 – 6.8 Kg.^33^ Eksovest is flexible and modular and is available in 3 different sizes and, hence, can be adjusted to users of different sizes, heights, and shapes.

#### LegX

LegX, a lower body passive exoskeleton, developed by SuitX is suitable for virtual chair and deep knee positions. It has lock and unlock modes for different positions. Lock mode has two mechanical functions that can help to sit on chair and knee position. The lock mode provides extra security to the user and the exoskeleton is locked to the desired position. At unlock mode, the user can move around freely while wearing the exoskeleton.

### Tasks description and sequence

**Figure 2.**
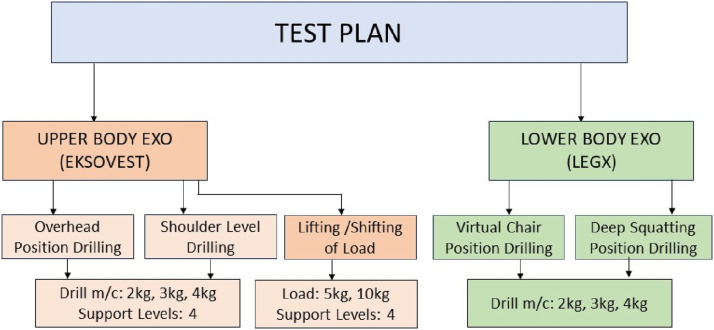
Flow chart of test plan.

**Figure 3.**
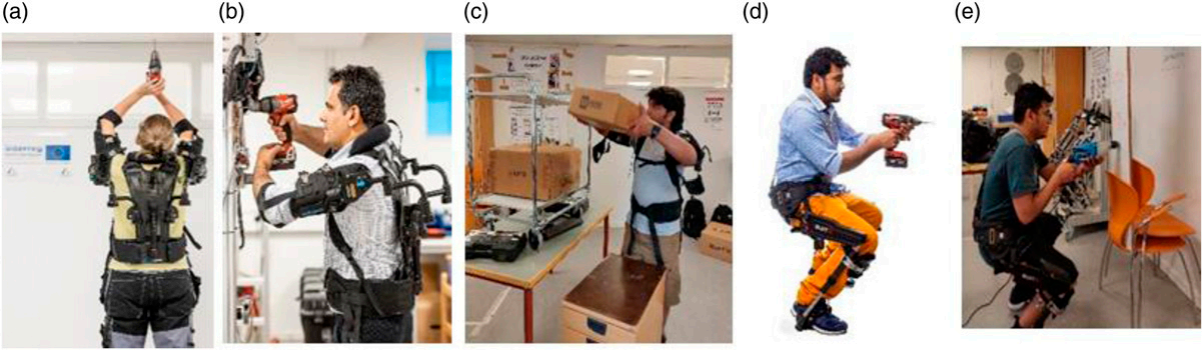
(a) Drilling at overhead position, (b) Drilling at shoulder level, (c) Lifting and shifting of boxes, (d) Wall drilling at virtual chair position and ( e) Wall drilling at deep squatting position.

Figure 2 below shows flow chart of the tests performed in this study. Whereas, Figure 3 shows different positions of workers’ while performing the designated tasks.

All participants performed all of the tasks mentioned below and shown in Figure 3, for all the cases of payloads and the support levels of the exoskeletons. The summary of the tasks performed are shown as below,

• stand and hold the drill machine, with and without wearing Eksovest, and perform drilling in the roof position using different drill machines having weights 2 kg, 3 kg and 4 kg. Different weights of drill machines were used to assess the relation between changes in muscle activity and level of assistance provided by the exoskeleton.
• drill in the wall at shoulder level with three different drill machines.
• lift and place different loads from one place to another position, with and without wearing Eksovest. The weight of the lifted boxes is 5 kg and 10 kg.
• virtual chair position tasks were performed, with and without wearing LegX. The participants drilled holes in a wooden board using three different drill machines (of weight 2 kg, 3 kg and 4 kg). In virtual chair position, the angle between the thigh and shank was 107.5^0^.
• deep squatting position tasks were also performed using LegX. The angle between the thigh and shank was 65.2^0^.

### Participants description and ethical approval

Several participants from different SMEs, logistics and construction companies participated in the workshops arranged as part of the EXSKALLERATE project. The participants put on the exoskeletons and observe the difference while performing different tasks of workers. However, for the data acquisition and analysis purposes, 9 volunteers were selected based on diversity in their height, weight, and body size. Seven of them were males and two participants were females. This physical diversity also ensured the effectiveness and usability of exoskeletons for the users of different physiques as shown in Figure 3. The ages of the participants were between 27 to 33 (mean 30 ± 3) years. The weights of the subjects were between 68 kg to 90 kg, and their heights were between 160 cm to 185 cm.

Prior to participating in the tests, all volunteers provided informed consent and confirmed that they had no signs of orthopedic or neurological issues. The study was conducted in accordance with the protocol approved by the Ethics Committee of “Regionala Etikprövningsnämnden Uppsala (Uppsala Regional Ethics Review Board)” (2012-03255).

### Data acquisition and data processing

Shimmer EMG sensors were used to record the muscle activity. The sensors were attached to the muscles where the possibility of maximum contraction or extension of muscles may occur. The data acquisition software, ConsensysPro (available by the Shimmer EMG sensors) was installed on the laptop which facilitated the data transfer from the sensors to the laptop wirelessly through Bluetooth. There are five ports on the Shimmer3 EMG sensor kit. The four ports are used to measure the muscle activity, whereas the fifth port is used to record the signal from the reference point. The reference point can be chosen as the place of minimum muscle activity, such as nearby bone or joint. All of the five ports are connected through surface electrodes to obtain the EMG signals.

**Figure 4.**
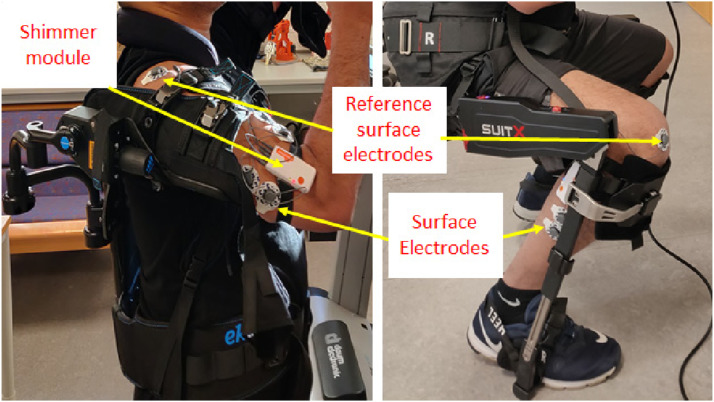
Placement of surface electrodes at muscles and bones.

**Figure 5.**
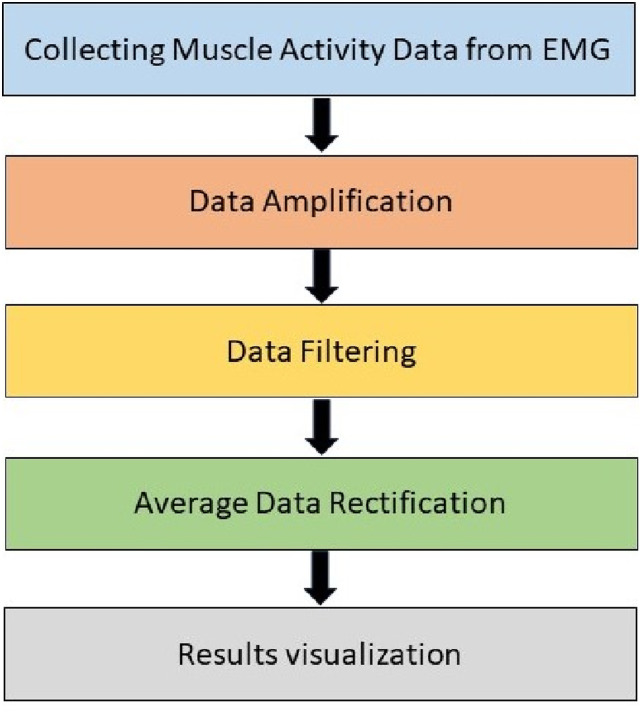
Block diagram for data processing.

Shimmer sensor kit was connected through surface electrodes, which were placed on the surface of the skin, just above the muscles of significance activity. These surface electrodes composed of Silver-Silver Chloride (Ag/AgCl). The electrodes were surrounded by an electrolyte or body electrode gel with a resistance of 100 Ohm. This gel is known as a non-irritating gel. The diameter of the surface electrode is 25 mm. Surface EMG electrodes (500x Covidien ECG Electrodes with Hydro Gel + Foam/57 x 34 mm) were placed on the biceps/triceps, thighs, and calf muscles for measuring muscle extension and contractions as shown in Figure 4. 1024 data readings were collected per second and a zero-lag, second-order Butterworth filter (frequency range 20–400 Hz) was used. The raw EMG data was bandpass filtered to eliminate movement irregularities and high-frequency noise. The ac interference was removed using a 20 Hz zero-lag second-order infinite impulse response high pass filter. The EMG signals were then rectified, and the envelope of the signal was calculated using a zero-lag lowpass filter having an envelope of 5 Hz. Lastly, the post-processing of the data and the graphs generation were performed in MATLAB R2021a. The sequence of data acquisition to data processing is shown in Figure 5.

To enhance the accuracy of the muscle activity signal, the reference electrode should be placed at an electrically neutral point of the body, as far away as reasonably possible from the muscle being measured. Bony prominences such as those at the shoulder bone were selected as a reference point. Firstly, EMG signals of each participant were recorded without using the exoskeleton to perform the selected task. This provided a baseline for comparison of how much reduction in the muscle activity could be achieved when the same task was performed with the exoskeleton by each participant.

EMG sensors recorded the Biceps and Triceps muscles activities as these muscles have been predominantly involved in the upper body tasks performed in this study. For example, to raise the forearm, the biceps contract and the triceps relax. To lower the forearm again, the triceps contract and the biceps relax. Furthermore, it was convenient to install the EMG toolkit and surface electrodes on these muscles and reference bony prominence location.

The sequence of data collection and data processing is presented in Figure 5.

Following mathematical relation was used to calculate the percentage reduction in human muscle activity, with and without wearing exoskeleton,

(1) $$\eta_{percentage\;reduction}=\frac{WOE-WE}{WOE}\times 100\%$$

Where, “WOE” represents measured values without wearing exoskeleton and “WE” are measured values while wearing exoskeleton.

## Results and discussion

The results of the study are demonstrated in this section. The results are presented for all the five tasks illustrated in Figure 3(a) to Figure 3(e). Three upper body tasks shown in Figure 3(a) to Figure 3(c) were performed using Eksovest. The Eksovest offered 4 assistance levels, namely Support Level 1 to 4, where 4 represents the highest support level and 1 provides the least assistance.

### Results of the upper body tasks

**Figure 6.**
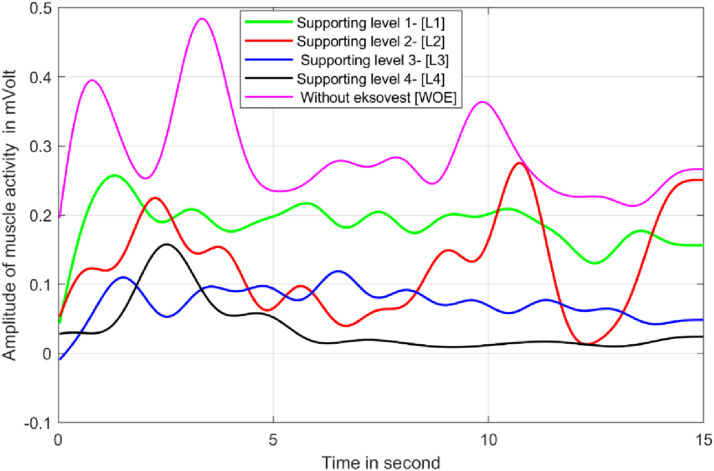
Muscle activity for overhead drilling task for 4 different level of support of Eksovest.

Figure 6 shows the average muscle activity of all participants for the overhead drilling task as depicted in Figure 3(a). In Figure 6, magenta line shows muscle activity without wearing exoskeleton and the green line represents muscle activity while wearing Eksovest at maximum support level (level 4). The black, blue and red color lines represent muscle activities at support Level 1, 2 and 3 of the Eksovest. It can be observed that the muscle activity of the workers was maximum while performing the overhead drilling task without wearing the Eksovest. Muscle activity was reduced gradually as the assistance level of the Eksovest was increased from level 1 to level 4.

**Figure 7.**
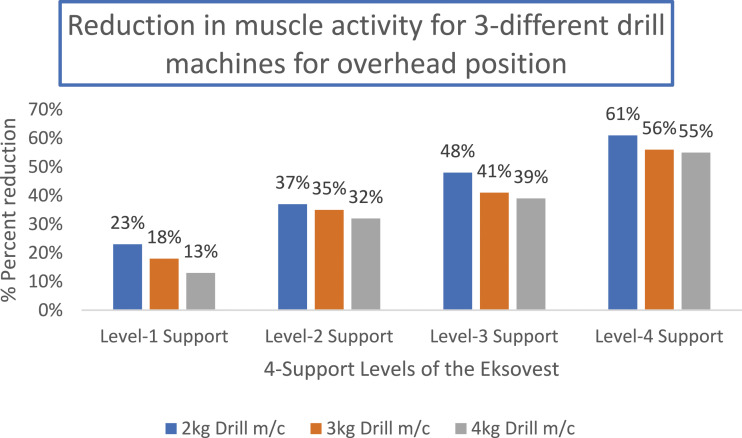
Percentage reduction in muscle activity for overhead position tasks.

Figure 7 shows the percentage reductions in muscle activities for each level of support of Eksovest for overhead drilling tasks using three drill machines of weight 2 kg, 3 kg and 4 kg.

**Figure 8.**
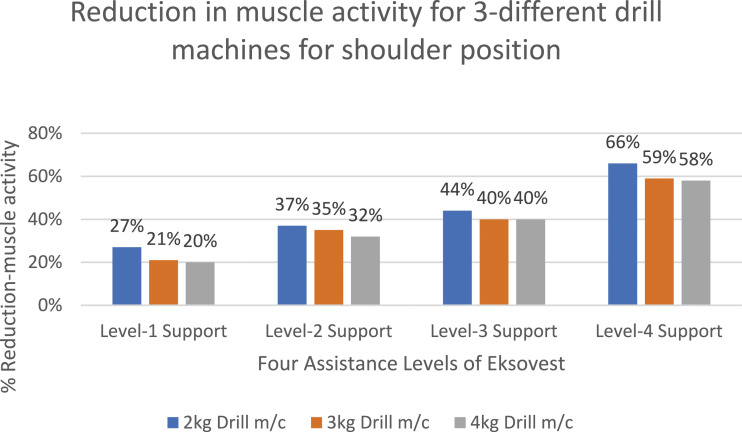
Percentage reduction in muscle activity for shoulder position tasks.

**Figure 9.**
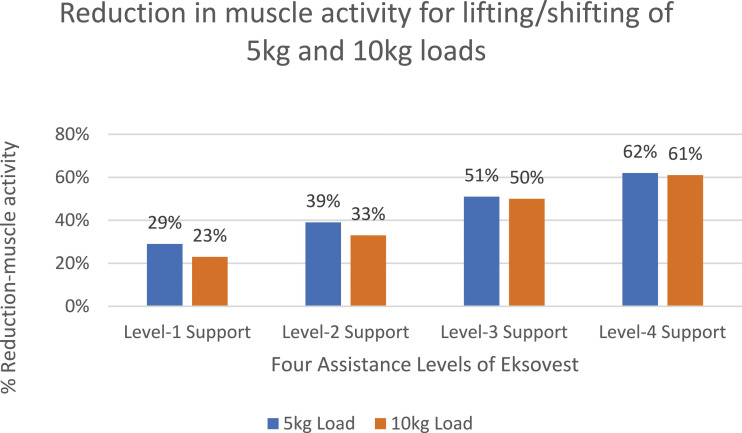
Percentage reduction in muscle activity lifting/shifting of loads (5 kg and 10 kg) from 90 cm to 170 cm high table/rack.

Similarly, Figure 8 and Figure 9 shows the percentage reductions in muscle activities for shoulder level drilling and lifting/shifting of two loads of 5 kg and 10 kg, respectively. These tasks correspond to the tasks shown in Figure 3(b) and (c). It is important to note that the reduction in muscle activities shown in Figure 7 to Figure 9 and Figure 11 are calculated using equation (1) of Section 2.4.

### Results of the lower body tasks

**Figure 10.**
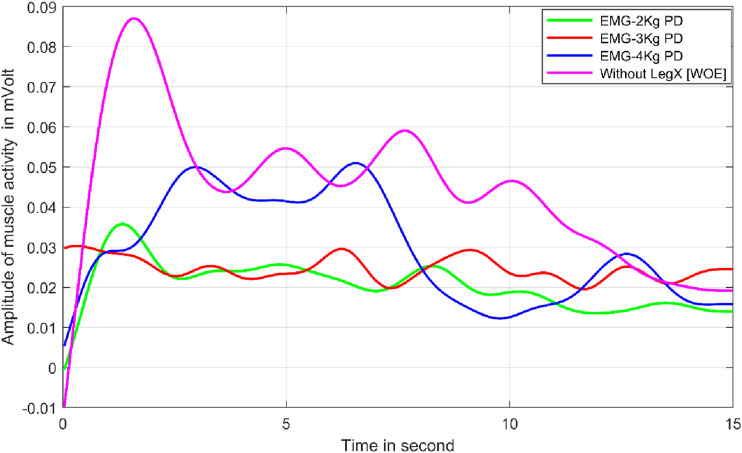
Muscle activity for virtual chair position wall drilling with and without exoskeleton.

As shown in Figure 3(d)-(e), two lower body tasks were performed using LegX exoskeleton. The two tasks were performed with the LegX exoskeleton (1) virtual chair position and (2) deep squatting or bending position. The workers working in these positions suffered commonly with knee and lower body injuries. Therefore, this study provides a viable solution to reduce stresses and loads on the lower body by using a suitable exoskeleton. Figure 10 shows the results of wall drilling in a virtual chair posture, as depicted in Figure 3(d).

The purple line shows the highest muscle activity as it corresponds to the drilling without exoskeleton (in virtual chair position), whereas the other color lines corresponds to the muscle activities while wearing LegX exoskeleton and operating drill machines of three different weights (2 kg, 3 kg and 4 kg). The muscle activity for all the tasks is plotted for 15 seconds for all the cases as the tasks were designed to complete in 15 seconds.

**Figure 11.**
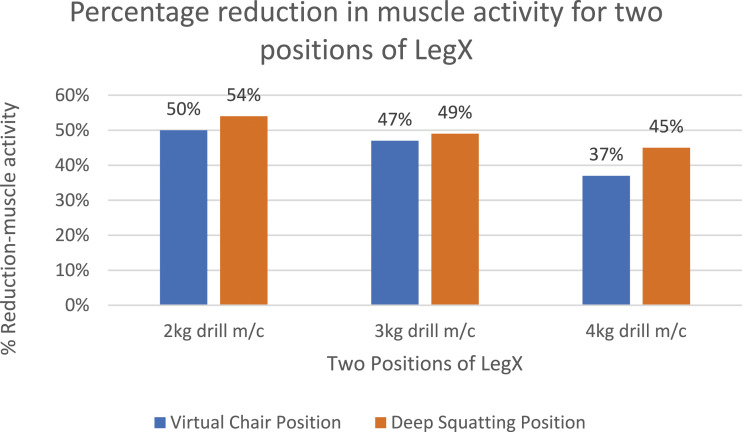
Percentage Reduction in muscle activity for virtual chair and deep squatting position tasks.

Figure 11 shows the average percentage reductions in the muscle activity of all workers while working in virtual chair and deep squatting position. It can be noted that a relatively less assistance is achieved while performing the lower body tasks using LegX. The results of Figure11 can be compared with the results of Figure 7 to Figure 9. This might be due to the fact that the upper body exoskeletons are easier to use, and no additional balancing is needed, whereas, the lower body exoskeletons has to bear and balance the load of the entire body and a slight misalignment can cause discomfort and balancing issues.

Figure 10 and Figure 11 presents the results of the lower body for virtual chair position and deep kneeling/squatting position. The thigh muscle activity was maximum when the participants did not use LegX for both positions while performing their tasks. Therefore, the study successfully demonstrated that all participants felt relieved from strains in the muscles with exoskeletons while performed the same tasks without wearing the exoskeletons.

### Limitations of the study

It is pertinent to mention that the muscle activity of the participants was affected due to the mounting location of the EMG package on the body as the mounting straps might exert pressure on muscles. This occurred as the length of the cables, connecting surface electrodes to the main EMG system was too small. Therefore, the location of surface electrodes and the mounting of the whole EMG system must be too close which might affect the muscle activity. It is recommended that the cable length should be large enough to mount the Shimmer EMG system away from the surface electrodes.

Secondly, the number of female participants available for this study was quite limited. A higher number of female participants having different ages and physiques would be more beneficial for future studies.

## Discussion and design recommendations

In the EXSKALLERATE project, more than 400 SMEs, construction and logistics companies participated in different events such as testing, seminars and workshops. Overall, the performance of more than 25 commercially available passive industrial exoskeletons was assessed, by various project partners. It was found that each of the exoskeletons tested was merely effective for a limited range of motion and for a specific posture. For all other postures and positions, the same exoskeleton was not helpful. The tests were conducted in a lab environment as well as pilot sites were set up at the actual premises of various SMEs, construction, and logistic companies to evaluate the performance in the real work environment.

Indeed, these exoskeletons provided useful support to certain muscles and reduced the muscle activity of the workers by up to 60%. However, there have been quite a few design issues which create usability problems which cause discomfort to the users. Therefore, based on the findings of this study, this section presents its recommendations to improve the design, usability features and comfort for the users. It is important to note that the design improvement recommendations presented in the below section have also been shared with the developers of LegX and Eksovest. The manufacturers appreciated the feedback of our experimental study and considered including it in their future versions. The summary of these design improvements are presented below.

### Emphasis on more realistic simulation

Even though the exoskeletons tested in this project provided assistance and support, still these devices are predominantly rigid and heavy, having poor human-exoskeleton interfaces, and are less compliant. Therefore, there is a dire demand to develop several simulation models before the development of physical prototypes since developing these physical prototypes utilizes a lot of time, labor and financial resources. Therefore, developers need to perform co-simulation between digital human and exoskeleton models.

### Biomechanical compatibility

Exoskeletons must be designed with a human-centered design process to achieve maximum acceptance. If biomechanical compatibility is not ensured, several unwanted consequences in the physical human-machine interaction happen. The most common shortcoming in the design of the existing exoskeletons is that human-rotation joints are modeled as hinge joints. For example, it was found in this study that the knee joint in the lower-body ‘LegX’ exoskeleton and the elbow joint in the upper-body ‘Eksovest’ exoskeleton, are simple hinge joints whose axis of rotations were fixed. Whereas, the human knee and elbow joints are complicated with varying rotation axis over flexion/extension. Therefore, the developed exoskeletons based on such a design where the rotation of human joints (e.g. knee, elbow) is assumed as a hinge will have misalignments between the joints of the physical prototype and the human user and will cause severe discomfort. This is an important example of how biomechanical modeling ought to be refined to deliver productive input to exoskeleton developers to avoid such misalignments.

### Enhancing flexibility and range of motion

**Figure 12.**
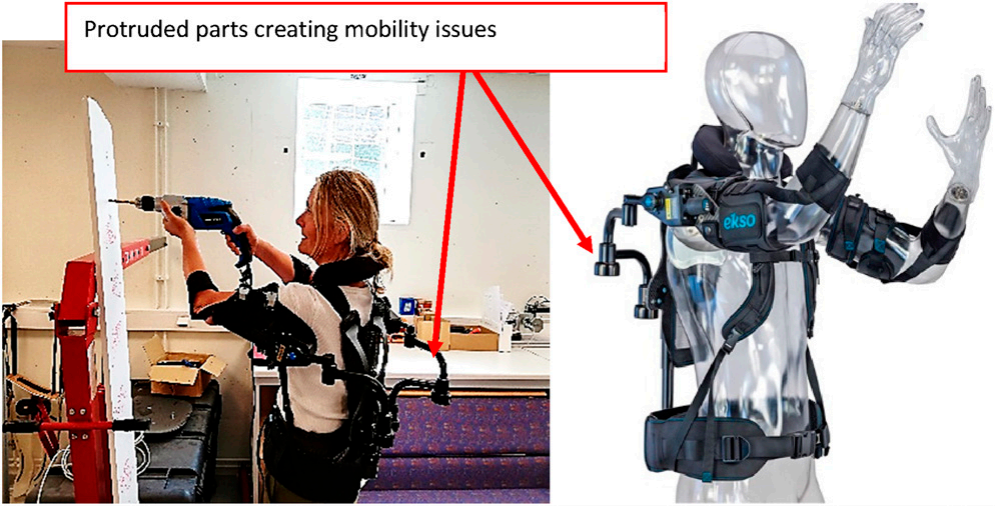
Testing of Eksovest.

The existing models lack inclusion of actual work-space related limitations, e.g., long protruded parts, as shown in Figure 12 below, exists in Eksovest restricts the mobility of workers while operating in narrow paths. Therefore, certain tools must be developed to simulate human-exoskeleton along with their work-space boundaries.

### Standardization and planning challenges

Lack of specific exoskeleton standards and certifications have been described as barriers to the acceptance of exoskeleton technologies in the industry. While exoskeletons are not considered a conventional form of personal protective equipment (PPE), though, they are similarly wearable and their main motivation is also to prevent work-related injuries.^41,42^ The standardization of industrial exoskeletons can be achieved by directly communicating to standards developing bodies, specifically ISO, CEN and their members. Subsequently, awareness about standardization can be improved by sharing the information with end-users, manufacturers, and distributers of industrial exoskeletons. One of the important objectives of EXSKALLERATE project was to coordinate with national standardization organizations of various EU countries to accelerate the standardization for exoskeletons. During this study, valuable feedback was also sought from the Swedish Institute of Standardization (SIS) regarding developing and implementing relevant standardization practices.^43^

## Conclusion

In this study, it was found that the passive exoskeletons significantly reduced muscle activity while performing workers’ tasks for overhead, shoulder, prolonged virtual chair, knee bending positions, and lifting/shifting of loads. The results showed a remarkable reduction in human effort, thereby, demonstrating a potential solution of reducing work-related MSDs. For the upper body tasks, as much as 66% reduction in muscle activity was observed while performed the same tasks without using the exoskeleton. For the lower body positions, the reduction in muscle activity was up to 54%. This is also quite significant reduction in human effort, however, further reduction in muscle activity can be achieved by practice and balancing of LegX. It was observed that while performing the prolonged virtual chair position and kneeling position tasks, the users not only have to correctly position the lower body but also have to properly balance the upper body as well. Due to these reasons, it was not easy for the users to use LegX comfortably. Based on the outcomes of this study, various design improvement strategies have been presented and also shared with the respective manufacturers of these exoskeletons. Although, a notable support was provided by these exoskeletons, still there exists significant design challenges which must be addressed.
